# Analysis of Factors Influencing the Decision-Making Behavior of Beef Cattle Farmers: An Empirical Analysis Based on Logit-ISM Model

**DOI:** 10.3390/ani12243470

**Published:** 2022-12-08

**Authors:** Xujun Li, Hao Zhang, Mingli Wang

**Affiliations:** Institute of Agricultural Economics and Development, Chinese Academy of Agricultural Sciences, Beijing 100081, China

**Keywords:** beef cattle farmers, cow breeding, decision-making behavior, influencing factors, Logit-ISM model

## Abstract

**Simple Summary:**

In recent years, the shortage of cow stocks and the reduction of cow breeders in China have caused a shortage of cattle resources and a high price of calves, which has become a bottleneck restricting the further development of China’s beef cattle industry. At present, there are few studies on the decision-making behavior of cow breeding in China. In order to explore the factors that affect the decision-making behavior on cow breeding by beef cattle farmers, this paper uses field survey data to make an empirical analysis through the Logit ISM model. The results show that the age of the breeder, the breeding year, the planting area of forage, market expectations, the loan amount, the breeding method policy publicity and policy subsidies are significant factors affecting the decision-making behavior of beef cattle farmers on cow breeding.

**Abstract:**

The beef cattle industry is an important part of herbivorous animal husbandry and an important industry to enrich the dietary structure of residents and promote the income of farmers and herdsmen. The cow is an important foundation to support the healthy development of the beef cattle industry, which is related to the stability of cattle sources and the sustainable development of the industry. However, in recent years, the shortage of cows in our country has led to the shortage of cattle resources and the high price of calves, which has restricted the further development of our beef cattle industry. In order to explore the factors that affect the decision-making behavior behind cow breeding and to find the right policy to mobilize the enthusiasm for cow breeding, based on the field survey of five provinces (cities and regions), including Inner Mongolia Autonomous Region and Ningxia Hui Autonomous Region, this paper conducts an empirical analysis through the Logit ISM model. The results show that market expectation is the surface factor that directly affects the decision-making behavior of cow breeding. The influencing factors of the middle layer are policy propaganda, policy subsidies, breeding years, and feeding methods. The deep root factors are the age of the breeder, the amount of loan, and whether to plant feed crops. Based on this, this paper proposes that we should actively promote the importance of cow breeding, strengthen policy support for cow breeding, formulate scientific and reasonable subsidy policies for cows, innovate financial support methods to solve the problem of fund shortage of farmers, and develop forage resources and reduce the breeding cost of cows.

## 1. Introduction

With the improvement of living standards and income levels of Chinese residents, consumers’ consumption concepts and dietary habits have changed greatly, and the consumption of animal foods with high protein and low-fat content has been increasing. The per capita pork consumption has declined, while the consumption of beef and lamb is increasing year by year [1]. The per capita apparent beef consumption in China was 4.20 kg in 2000, which increased to 6.26 kg in 2020, an increase of 49.05%. The beef cattle industry has become an important part of China’s livestock industry and has played an important role in adjusting and optimizing the dietary structure to promote farmers’ income and develop the rural economy [2]. However, in recent years, the domestic beef supply is increasingly tight, beef imports continue to increase, and in 2020 China’s beef imports reached 2.12 million tons, accounting for about 24% of total domestic beef consumption, and China has become the world’s largest beef importer. In order to meet domestic beef consumption demand and enhance domestic beef supply capacity, it is imperative to accelerate the development of the beef cattle industry. Cow breeding is an important foundation for the healthy development of the beef cattle industry, which is related to the stability of cattle sources and the sustainable development of the beef cattle industry [3].

Since entering the 21st century, there has been a sharp decline in China’s breeding females, and the shortage of cow resources has led to a rapid rise in calf and rack cattle prices, increasing the cost of beef cattle farmers and shaking the foundation of the development of the beef cattle industry [4]. In order to enhance the enthusiasm for farmers’ cow breeding, in 2014, the General Office of the Ministry of Agriculture and Rural Affairs and the General Office of the Ministry of Finance launched the beef cattle basic cow expansion project in 15 major producing provinces and regions, and the central government arranged about 130 million dollars of funds to implement subsidies, focusing on mobilizing the enthusiasm of raising cows. Under the guidance of this project and the high market price of beef cattle in recent years, the stock of basic beef cattle in China has recovered to a certain extent, but farmers are still not motivated to breed cows, and the lack of stock of cow is still an important factor limiting the development of China’s beef cattle industry.

The objective of this study was to determine why it is difficult to mobilize enthusiasm for cow breeding, to further explore the factors that affect the decision behavior of cow breeding by farmers, systematically analyze the factors that affect the decision behavior of cow breeding from different angles such as economy, society, and policy, and identify the hierarchical relationship between the factors. So as to provide relevant references for stabilizing the cattle source, mobilizing the enthusiasm for cow breeding, and ensuring the sustainable development of China’s beef cattle industry and the supply capacity of beef products.

## 2. Materials and Methods

### 2.1. Data Sources

The data source used in this paper is the field survey data of five provinces (cities and districts) in the Inner Mongolia Autonomous Region, Ningxia Hui Autonomous Region, Sichuan Province, Chongqing Municipality, and Shanxi Province during September-November 2021 by the subject group. These five provinces (regions and municipalities) are significantly representative of typical provinces of the northeast production area (Inner Mongolia Autonomous Region), northwest production area (Ningxia Hui Autonomous Region), southwest production area (Chongqing City and Sichuan Province) and central plains production area (Shanxi Province) of beef cattle production, respectively. A total of 317 valid questionnaires were returned, the respondents are generally farmers or producers, which can better reflect actual production. These five provinces are distributed in four major beef cattle-producing areas in China, and their beef production is at the forefront, which can better represent the overall situation of beef cattle breeding in China. The specific regional distribution and sample size are shown in Table 1.

### 2.2. Variable Selection

Explained variables: the explanatory variable being solved is the decision behavior of whether to breed cows or not, with 1 indicating yes and 0 indicating no.

Core explanatory variables: The core explanatory variables include the breeder’s age, education level, training received, years of breeding experience, feeding method, whether or not forage crops are grown, loan amount, whether or not someone in the household is a village level or higher cadre (including village-level cadres), whether or not they are a member of a farmers’ cooperative/association, market expectations, policy advocacy, policy subsidies, and calf racking cattle prices.

Control variables: In order to reduce the influence of geographical location conditions and some unobservable variables on the decision-making behavior of cow breeding, regional dummy variables were set with reference to existing studies [5]. The definition and descriptive statistics of each variable of the model are shown in Table 2.

### 2.3. Binary Logit Model

Since whether to breed cows or not is a typical binary decision model, this paper uses a binary logit model to analyze each influencing factor, and the model is set as follows.

(1) $$P=F\left(y=1|x_{n}\right)=\frac{1}{1+e^{-y}}$$

The equation can be converted as follows

(2) $$\ln\left(\frac{p}{1-p}\right)=\beta_{0}+\beta_{1}x_{1}+\beta_{2}x_{2}+\ldots \beta_{n}x_{n}+\mu$$

where *P* represents the probability of beef farmers’ decision to breed cows, and $x_{n}$ is the explanatory variable, which consists of influencing factors such as personal characteristics, environmental characteristics, and cognitive characteristics of farmers, and β is the parameter to be estimated, and μ is the random error term.

### 2.4. The Explanatory Structure (ISM) Model

The Interpretative Structural Model (ISM) was first proposed by Warfield. The model is usually applied to study the internal structure and hierarchy of a system. The core of the model is to decompose a complex system into several subsystems, analyze the correlation of each subsystem according to the principle of correlation matrix, and then analyze the logical relationship and hierarchy between the subsystems [6]. The factors that affect farmers’ cows are both independent of each other and influence each other. Based on this, the ISM model is used in this study to explore the logical relationships among the influencing factors. The specific steps are as follows.

In the first step, the logical relationships among the influencing factors were judged and the adjacency matrix was constructed. Based on the regression results of the logit model, the factors influencing the decision to breed cows were determined as $S_{i}\left(i=1,2,3\ldots n\right)$, denoted by $S_{0}$ denotes the behavioral choice of farmers’ cow breeding decisions. The logical relationship between factors refers to whether there is a direct “interaction” or “mutual premise” relationship between any two factors, including $S_{I}$ with $S_{0}$ and $S_{i}$, the relationship between the two factors. The adjacency matrix is obtained from the logical relationship between the factors. The components of the adjacency matrix(R) are defined as follows.

(3) $$r_{ij}=\{\begin{array}{l} 1,s_{i}\;is\;related\;to\;s_{j}\;\\ 0,s_{i}\;is\;not\;related\;to\;s_{j}\; \end{array}$$

Among them i=1,2,3…n; j=1,2,3…n.

In the second step, the reachable matrix is calculated, using Equation (4) to calculate the reachable matrix.

(4) $$M={\left(R+I\right)}^{\lambda +1}={\left(R+I\right)}^{\lambda }\neq {\left(R+I\right)}^{\lambda -1}\neq \ldots \neq {\left(R+I\right)}^{2}\neq (R+1)$$

where *I* is the unit matrix 2≤λ≤n and the Boolean operator is used for the power operation of the matrix.

In the third step, the reachable matrix M performs area decomposition and interlevel decomposition. That is, determine the M, the reachable set $P\left(s_{i}\right)$, and the prior set $Q\left(s_{i}\right)$ where the reachable set $P\left(s_{i}\right)$ denotes the reachable matrix M element of the matrix $s_{i}$ of the matrix elements with “1” in the corresponding row. The prior set $Q\left(s_{i}\right)$ denotes the set of elements of the reachable matrix M elements of the matrix $s_{i}$ The set of row elements corresponding to the matrix elements containing “1” in the corresponding column. The hierarchy is determined as follows.

(5) $$L=\{s_{i}|P(s_{i})\cap Q(s_{i})=P(s_{i})\}$$

in the form of i=1,2,3…,n. According to Equation (5), we can determine the highest level and the highest level $L_{1}.$ The set of elements in the highest and highest levels can be determined, and then the elements contained in the other levels can be determined in order from high to low. The other levels are determined by deleting the original reachable matrix M in $L_{1}$ to obtain the reachable matrix $M_{1}$. According to formula (5), we calculate $M_{1}$, the highest level of the $L_{2}$, and so on to the set of elements at the bottom level.

In the fourth step, the explanatory structure model is built. The hierarchical structure among the factors influencing farmers’ ewe breeding decisions is obtained by connecting the factors in the same and adjacent levels with directed edges.

## 3. Results and Analysis

### 3.1. Analysis of Factors Influencing the Decision-Making Behavior of Beef Cattle Farmers’ Cow Breeding

The ordinary least squares regression (OLS) was performed on the model before conducting the regression considering that using a binary explanatory variable of type 0–1 would cause the model to lose some information as a control. The regression results are shown in Table 3. Model 1 is the result of regression with all independent variables included in the Logit model, and Model 2 is the result of final regression after excluding insignificant independent variables. Model 3 and Model 4 are the results of OLS before and after excluding the insignificant independent variables, respectively. It can be seen that the estimation results of the Logit model and the OLS model are basically consistent in terms of the significance of the variables, which also indicates that the estimation results of the Logit model are robust.

The results from the regression of Model 2 show that the eight variables of breeder’s age, breeding years, feeding method, whether to grow forage crops, loan amount, market expectation, policy publicity, and policy subsidy passed the significance test. Among them, the farmer’s age has a negative effect on the decision-making behavior of beef cattle farmers in raising cows at the 10% statistical level, that is, the older the farmer, the lower the probability of choosing to raise cows, mainly because cow farming requires higher requirements for farming technology and labor intensity, which requires more energy and labor, in addition to the long cycle of cow farming, which is greatly affected by uncertainties such as market conditions and higher farming risks The older the farmer, the less likely he is to choose to breed cows.

The number of years of breeding has a positive influence on the decision-making behavior of beef cattle farmers in breeding cows at the 1% statistical level, i.e., the longer the number of years of breeding beef cattle, the higher the probability of farmers choosing to breed cows, mainly because the long years of breeding reflect that farmers are optimistic about the long-term development situation of the beef cattle industry. And the longer the breeding years, the richer the experience and technology of beef cattle breeding, which can improve the production performance of cows, and reduce the risk of cow breeding, as well as the relatively small obstacles to engaging in cow breeding. The longer the farming years, the greater the possibility of continuing to engage in beef cattle breeding. As an important basis for the development of the beef cattle industry, farmers who have been engaged in beef cattle breeding for a long time also have a deeper understanding of the importance of beef cattle breeding, and the higher the probability of making decisions about beef cattle breeding.

The farming method had a positive effect on the decision-making behavior of beef cattle farmers’ cow breeding at a 1% statistical level, indicating that the probability of choosing cow breeding was higher for the farmers who chose free-range and semi-herded semi-herded. Due to the long breeding cycle and high feed cost of cows, the farmers who free-range or semi-herding are able to use pasture to reduce the input of certain feed costs; and free-range can increase the movement of cows, which is conducive to improving the performance and animal welfare of cows [7]; free-range can also save certain labor cost and labor time.

Whether or not to grow forage crops has a positive effect on beef cattle farmers’ cow breeding decision behavior at the 1% statistical level, i.e., farmers who grow forage crops have a greater probability of breeding cows. Due to the rapid increase in the price of fine and roughage feeds in recent years, which seriously increases the cost of beef cattle breeding [8], cows have a long breeding cycle and consume much more forage than the short-term fattening model. According to the research data, the current full self-breeding households need to put in more than 625 dollars for a 500 kg beef cattle, accounting for about 40% of their total costs, farmers who grow their own forage can largely reduce the breeding by directly increasing breeding profits, reducing the financial pressure brought about by the long cow breeding cycle; it can be said that forage planting is one of the key factors in determining the cow breeding.

The amount of loan has a negative effect on the behavior of beef cattle farmers’ cow breeding decisions at the 1% statistical level, i.e., farmers with more loan amounts are less likely to breed cows. This is mainly due to the fact that farmers with higher loan amounts bear higher amounts of interest. The cow breeding cycle is long and capital turnover is slow, so the larger the loan amount, the more farmers tend to choose the short-term fattening model with a fast turnover and less capital pressure.

Market expectations have a positive effect on beef cattle farmers’ cow breeding decision behavior at the 1% statistical level, i.e., the more confident farmers are in the industry and the market, the more bullish they are about expected returns, the greater the probability of breeding cows.

Policy advocacy has a positive effect on the decision-making behavior of beef cattle farmers for cow farming at the 1% statistical level, indicating that the importance of promoting cow farming and encouraging farmers to raise cows can increase the motivation to raise cows. Moreover, in general, places where the government actively promotes the breeding of cows tend to have supporting policies to encourage cow breeding, which can increase the motivation of farmers to breed cows.

The availability of local subsidies for cow breeding has a positive impact on farmers’ decision-making behavior for cow breeding at a 5% statistical level. Since the current national level cow expansion and increase, the project has basically stopped. On the local level, only some local governments have introduced and implemented the cow expansion and increase project, and the number of subsidies vary from region to region. In these cases, regions with policy subsidies have relatively higher cow breeding. The probability of choosing to breed cows is also greater than in places without subsidies for cow breeding.

### 3.2. Hierarchical Relationship Analysis of Factors Influencing the Decision-Making Behavior of Beef Cattle Farmers’ Cow Breeding

**Figure 1:**
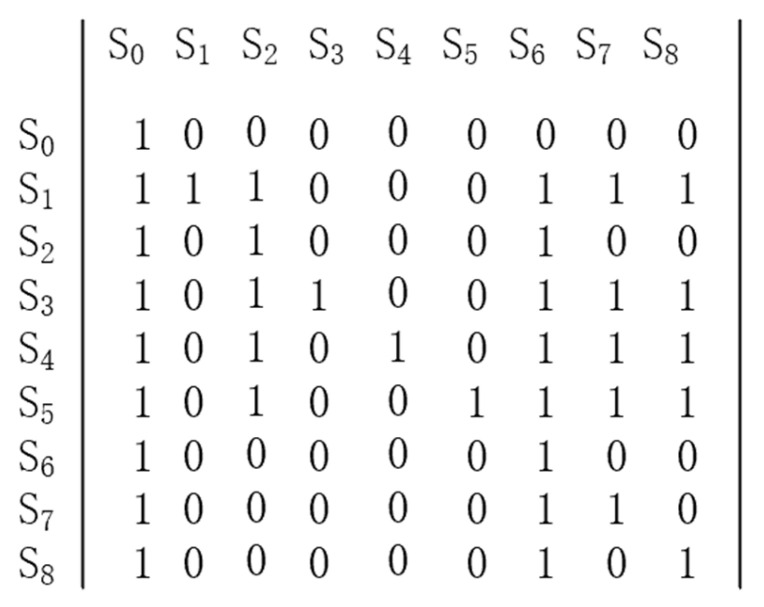
Reachability matrix.

**Figure 2:**
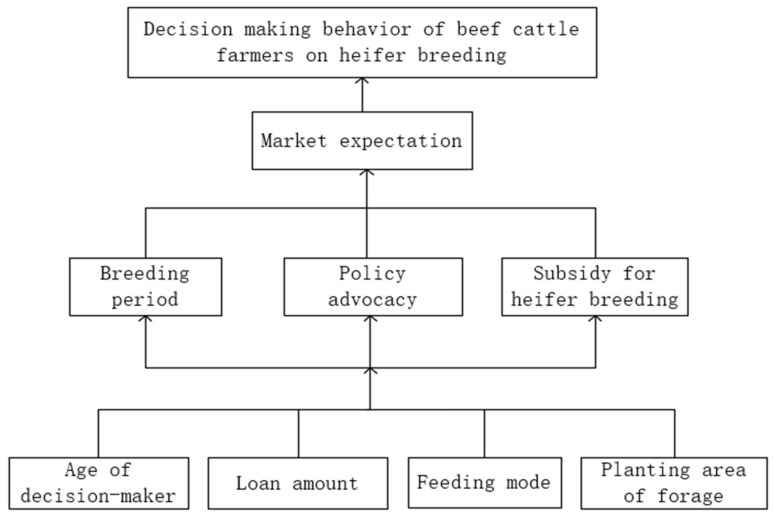
Hierarchical structure diagram of influencing factors.

The estimation results of the above logit model showed that eight influences passed the significance test, using S0 to denote the decision-making behavior of beef cattle farmers to breed cows and S1, S2, S3, and S8 in order to denote the eight dominant influencing factors. Based on the reference of existing studies, the logical relationship between the eight influencing factors was firstly determined, and the logical relationship between beef cattle farmers’ cow breeding decision and its influencing factors was constructed based on Equation (4). Then, the reachability matrix was obtained according to Equation (5), the reachability matrix is shown in Figure 1. Finally, according to the reachability matrix, the set of factors contained in each layer was determined by Equation (6) as $L_{1}=\left\{S_{0}\right\}$, $L_{2}=\left\{S_{6}\right\}$, $L_{3}=\left\{S_{2},S_{7},S_{8}\right\}$, $L_{4}=\left\{S_{1},S_{3},S_{4},S_{5}\right\}$. Finally, according to the hierarchical relationship, the factors in the same stratum and adjacent strata were connected by directed edges to obtain a hierarchical structure diagram of the factors influencing the decision-making behavior of beef cattle farmers’ cows (Figure 2).

The mechanism of occurrence of beef cattle farmers’ cow breeding decision behavior is that the surface layer factors are the direct drivers of farmers’ cow breeding decision behavior, and the intermediate layer indirect factors finally act on the target layer by indirectly influencing the surface layer factors, and the deep root factors are the most fundamental reasons affecting farmers’ cow breeding decision. According to the results of ISM analysis of beef cattle farmers’ cow breeding decision-making behavior, it can be seen that the surface layer direct drivers affecting beef cattle farmers’ cow breeding decision-making behavior are farmers’ expectations of the market situation of the beef cattle industry and cow breeding, that is, economic benefits are the surface layer factors affecting their breeding decisions, while breeding years, policy propaganda, cow subsidies, and feeding methods are the factors affecting market expectations, and thus the final decision. The middle layer factors and the bottom layer factors that influence farmers’ cow breeding decisions are the most basic resource endowment levels such as farmers’ age, loan amount, and whether they grow forage crops. The resource endowments possessed by beef cattle farmers are catalyzed by the intermediate layer factors which in turn influence the market expectations and ultimately influence farmers to make different decisions on cow breeding.

Among the tabular factors, the coefficient of influence of market expectations was 1.368, which had a positive effect on the decision-making behavior of beef cattle farmers’ cows at a statistical level of 1%. For the nonlinear model, the regression coefficients have limited economic significance [9], so focusing on the sign and significance of the parameters, it can be seen that the direction of influence of market expectations is consistent with expectations. Although cows are the basis for the sustainable development of the beef cattle industry, farmers’ behavioral choices generally follow the principle of profit maximization and always tend to favor profitable decisions [10]. Even if cow breeding is very important for the whole industry, it does not necessarily motivate farmers to make decisions on cow breeding, only when farmers believe that breeding cows can bring greater economic benefits can they be motivated to make decisions on cow breeding, and the better the market expectations for cow breeding, the higher the expected benefits farmers can obtain from breeding cows, and the more motivated they can be to breed cows.

Expectations of market conditions for cow breeding are in turn influenced by intermediate layer factors. The three intermediate layer factors, breeding years, policy advocacy, policy subsidies, and feeding practices, all positively influence the decision-making of cow breeding in the same direction as expected. The main reason is that farmers with longer breeding experience have relatively better expectations of the market, have confidence in the development of the industry, and regard it as a long-term business, not just looking at the amount of immediate profit, but considering sustainable development and valuing the stability of cattle supply. Policy propaganda will influence farmers’ judgment of the market and thus their expected returns; subsidies for cattle breeding make up for certain production costs and increase the economic benefits of cattle breeding; the cost of breeding varies depending on the feeding method, and farmers who are free-range or semi-herded are able to improve their breeding efficiency by saving on feeding costs.

The factors that affect the bottom of the cow farming decision are the age of the farmer, the amount of the loan, and whether or not to grow forage. Cow farming greatly differs from other farming practices in that cow farming does not focus on eating cows, the output of farmed cows is measured in calves, and in order to ensure the productivity of cows, the requirements for farming techniques are higher than ordinary fattening, and the cow farming cycle is long, usually, the reserve cows can only breed and become pregnant in the second year, calve in the third year, and calves can enter the beef market in the fourth year. The physical strength, energy, and technical acceptability of the breeding subject are higher, so the age of the breeder is a deep factor in deciding whether the breeder breeds cows or not; the long cycle of cow breeding, financial pressure, and feed costs are the main factors limiting the breeder’s entry into cow breeding, and if the breeder has a higher loan amount, the interest and financial pressure borne will affect his decision to breed cows; whether to plant forage crop, on the one hand, affects the feed cost of farmers, and on the other hand, it is also related to the problem of the consumption of manure from beef cattle breeding. From the root factors, it can be seen that what really affects the decision-making behavior of farmers’ cow breeding is the basic resource endowment of farmers, so the formulation of subsidy policies and the implementation of publicity and guidance should be formulated according to local conditions combined with the most basic resource endowment of farmers.

It is worth noting that according to the results estimated from Model 2, the constant term of the decision behavior of cow breeding is negative, indicating that without considering the influence of any factors, beef cattle farmers are reluctant to breed cows, and the possible explanation is that the large investment in cow breeding, long breeding cycle and slow return of benefits cannot stimulate a strong endogenous motivation of farmers, and without policy propaganda guidance and financial subsidies, many farmers without policy guidance and financial subsidies, many farmers are not highly motivated to breed cows.

## 4. Discussion

### 4.1. Similarities and Differences with the Existing Research

In order to explore the development path of China’s beef cattle industry, some scholars have conducted relevant studies. Wang Mingli et al. [11] analyzed the development situation of China’s beef cattle industry and concluded that the development of the beef cattle industry is a comprehensive system consisting of breeding females, calves, racking cattle, fattening cattle rearing, slaughtering and processing, storage and transportation, and marketing. In this production system, the quantity and quality of cattle supply is directly related to the rise and fall of the whole beef cattle industry; Wang Jiahuan [12], using research data from the dominant cow breeding regions in Jilin Province, concluded that opportunity cost is an important factor influencing the decision to breed cows, and the decrease in cow breeders is mainly due to the increase in urbanization and employment opportunities, and farmers give up breeding cows for more efficient industries; Ren Jizhou et al. [13] showed that the average annual growth rate of total breeding costs in China’s main beef cattle production areas reached 15.2% due to the significant increase in production costs such as labor costs, litter inputs, and feed costs; Ma et al. [14]. By analyzing the cost efficiency of different scales of beef cattle breeding in China, it was concluded that the prices of beef cattle breeding input factors have been on the rise in recent years, and the cost pressure on the beef cattle industry has further increased, with litter costs and feed costs rising. The cost pressure on the beef cattle industry has increased further in recent years, among which the cost of piglets and feed has increased especially.

In summary, existing studies have provided very valuable references for China to find the development direction of China’s beef cattle industry, but few studies have conducted a more systematic analysis of the decision-making behavior in cow breeding, and generally focus on the factors affecting farmers’ cow breeding decisions on economic benefits, however, the reality is that with the good development of China’s beef cattle industry in recent years, the economic benefits of cow breeding has improved, but the motivation toward cow breeding is still not high. This suggests that economic efficiency does not fully explain the motivation behind farmers’ cow breeding decision-making behavior. This study analyzed the factors influencing the decision-making behavior of cow breeding from different perspectives such as economic benefits and resource endowment, and found that the most fundamental factor affecting the decision-making behavior of cow breeding is resource endowment, which helps the Chinese government to formulate more reasonable industrial policies, better encourage farmers to raise cows, and thus ensure the sustainable development of the beef cattle industry.

### 4.2. Limitation and Future Research Direction

This study strives to accurately and objectively describe the influencing factors of cow breeding in China. However, since the data on cows raised by Chinese beef cattle farmers are not public, our research cannot be analyzed at the national level. We can only select several representative provinces through research to reflect the overall situation of cow breeding in China as much as possible, but there is still some unavoidable deviation, This is also the place that needs further exploration in the follow-up of this study.

## 5. Conclusions and Recommendations

### 5.1. Conclusions

In this paper, based on actual research data, the factors influencing the decision-making behavior of beef cattle farmers to breed cows and their recursive structure were explored using the Logit model and ISM analysis. The results of the study showed that whether beef cattle farmers choose to breed cows or not is the result of a combination of factors.

First, whether beef cattle farmers choose to breed their cows is significantly influenced by the breeder’s age, years of experience in breeding, whether they grow forage, expected returns, loan amount, breeding practices, policy advocacy, and policy subsidies.

Second, the eight dominant factors influencing beef cattle farmers’ cow breeding decisions are both independent and interrelated, and are at three different levels, among which, the market expectation is the surface-level influencing factor directly affecting beef cattle farmers’ cow breeding decisions; the intermediate level influencing factors are policy propaganda, policy subsidies and breeding years and breeding methods; the age of farmers, loan amount and whether to grow forage are the deep root factors.

### 5.2. Policy Recommendations

Based on the above research findings, considering the current reality of low motivation of beef cattle farmers in China to breed cow, insufficient cow stock, and shortage of cattle sources, the following recommendations are made to enhance the motivation of beef cattle farmers in China to breed cow and consolidate the sustainable development capability of China’s beef cattle industry.

Actively promote the importance of cow breeding and guide farmers to breed cows on an appropriate scale. On the one hand, through publicity boards, lectures, newspapers, video clips, and other means to promote the importance of basic cow breeding to farmers, the urgency of stable cattle sources, to raise awareness of the importance of cow breeding; on the other hand, to strengthen the publicity of relevant government support policies, to improve farmers’ knowledge of market expectations, to explain policies such as tax breaks, fee reductions, subsidies, interest subsidies and other policy content, so that farmers fully understand the relevant policy and in addition, through publicity and technical promotion, to guide farmers to develop a moderate scale of cow breeding. The current basic cow breeding farming mainly relies on small-scale free-range farmers, and there is still a gap between farming communities and large-scale farming. But cow breeding should not pursue too large, moderate scale family feeding model is in line with the laws of beef cattle breeding and Chinese characteristics of the model.

Strengthening the policy support for cow breeding and reasonably setting the subsidy standard is recommended. Since cow breeding is a basic industry related to the development of the whole beef cattle industry, it is a weak industry [15], which needs policy protection and support. The development of a reasonable subsidy standard can, to a certain extent, alleviate the pressure of production costs of farmers, improve the efficiency of cow breeding, and has a certain role in stimulating enthusiasm for cow breeding. At present, some local governments have implemented subsidies for cow breeding at the local level, but the subsidy standard is generally low due to the local financial level, which makes it difficult to mobilize the enthusiasm of farmers. It should be considered from the national level to continue to implement the basic cow expansion project, expand the amount of basic cow breeding according to the way of calving and supplementing the mother, and give subsidies for large-scale breeding to farmers whose cow breeding quantity reaches a certain scale. The implementation of different breeds of subsidies for cow farmers. This is not only conducive to stabilizing the stock of cows but is also beneficial for improving the level of good breeding of cows. It is also necessary to give special subsidies for cattle breeding facilities and equipment and other inputs, so as to promote and stimulate the moderate-scale operation of the cow breeding industry, at the same time improving the basic cow ID registration system out of the scientific and reasonable slaughter of breeding cows and reserve cows of the relevant provisions to limit and restrain the private slaughter of cows, and strive to make subsidies to cover every cow farmer. Subsidies should not only encourage the expansion of the number of cow breeding, but also encourage green breeding, and improve the sustainable development level of beef cattle breeding by means of subsidies for the resource utilization of feces and carbon trading.

Strengthening financial support to solve the problem of shortage of funds for farmers is recommended. There is a large one-time investment in cow farming, slow recovery of investment in the sudden characteristics of the normal financial environment, cow farming due to no collateral, risky often loan financing is difficult, even if the loan is raised, the long cycle of interest generated by the pressure is also very large. Therefore, policies at all levels can support policies through the development of cow breeding, loans for cow breeding to give discount interest, innovative financing methods, and financial institutions to provide convenient and flexible loan financing services for the cow breeding industry.

It is recommended to develop forage resources, encourage the combination of breeding and raising modes, and reduce the cost of cow breeding. Due to the special nature of cow breeding, it usually takes 3-4 years to raise cows in reserve to sell them after calving and fattening, so the high cost of feeding and the increased risk of breeding are the main factors leading to the low motivation of cow breeding. According to research data, feed costs account for about 30% of the total cost, and there is a rising trend year by year, and the rise in its price will inevitably affect the profits of the calf production chain. Therefore, in order to improve the economic efficiency of the beef cattle industry, the first thing is to control the cost of feed, and in the case that the land area for growing feed crops cannot be increased significantly, forage resources can be developed in a variety of ways, such as through strengthening technological research and development to improve the output per unit area; or make full use of crop straw, modulating and processing roughage through fermentation and other technologies to improve its digestibility and palatability; and also strengthen the forage Industry development, promote the planting of high-quality forage. At the same time, actively promote the combination of breeding modes, to achieve a positive interaction between planting and breeding, so as to reduce the cost of breeding and stabilize the sustainable and healthy development of the current cow breeding industry.

## Figures and Tables

**Table 1:** Regional distribution of the research sample.

Production Area	Representative Provinces	Representing Counties (Districts and Cities)	Sample Size
Northwest Region	Ningxia Hui Autonomous Region	Haiyuan County, Xiji County	143
Southwest Region	Chongqing	Hechuan District, Fengdu County	82
	Sichuan Province	Gyunlian County, Pingchang County	
Midland Region	Shanxi Province	Jinzhong City, Xiaoyi City	50
Northeast Region	Inner Mongolia Autonomous Region	Aruqorqin Banner, Bahrain Right Banner	42

**Table 2:** Definition and descriptive statistics of each variable of the model.

Variables	Variable Meaning and Assignment	Average Value	Standard Deviation	Expected Direction
Whether to breed cows	1 = yes 0 = no	0.86	0.35	/
Education level of decision-makers	1 = No formal education 2 = Elementary 3 = Junior high 4 = High school 5 = University 6 = Graduate 7 = Other	2.96	0.74	+
Age of breeder	Age of main breeder (weeks)	46.28	8.08	−
Have participated in cattle production skills training	1 = yes 0 = no	0.84	0.37	+
Years of breeding	Number of years in beef cattle breeding	10.59	8.96	+
Feeding method	1 = captive breeding 2 = stocking 3 = combination of captive and stocking	1.38	0.76	+
Whether to grow forage crops	1 = yes 0 = no	0.70	0.46	+
Loan amount	Average annual loan amount within the last three years (USD)	16,390.2	48,920.58	−
Whether anyone in the family is a village-level or above cadre (including village-level cadres)	1 = yes 0 = no	0.03	0.17	+
Whether a member of the farmers’ cooperative/association	1 = yes 0 = no	0.27	0.45	+
Market Expectations	1 = Very unpromising 2 = Less optimistic 3 = Generally 4 = More optimistic 5 = Very optimistic	2.87	0.96	+
Policy Advocacy	Has there been local publicity and encouragement for breeding cows 1 = yes 0 = no	0.83	0.37	+
Policy Subsidies	Are there local policies related to subsidies for cow breeding (feces recycling subsidies, calving subsidies, etc.) 1 = yes 0 = no	0.29	0.45	+
Calf racking cattle prices	Average selling price of calves and racked cattle (USD/kg)	7.07	1.19	+
Geographic location (control variable)	Northeast producing area = 1 Northwest producing area = 2 Southwest producing area = 3 Central producing area = 4	2.44	0.91	/

**Table 3:** Regression results.

Explanatory Variables	Logit Model 1		Logit Model 2		OLS Model 3		OLS Model 4	
	Coefficient	*p*	Coefficient	*p*	Coefficient	*p*	Coefficient	*p*
Education level of decision- makers	−0.244	0.553			−0.012	0.582		
Age of breeder	−0.093 **	0.019	−0.064 *	0.072	−0.004 *	0.052	−0.003	0.110
Training on cattle production skills	1.021	0.200			0.075 *	0.078	0.074 *	0.075
Years of breeding	0.266 ***	0.000	0.247 ***	0.000	0.003	0.178		
Feeding method	1.199 **	0.035	1.263 **	0.005	0.048 *	0.051	0.043 **	0.033
Whether to grow forage crops	3.363 ***	0.000	3.222 ***	0.000	0.256 ***	0.000	0.257 ***	0.000
Loan amount	−0.309 **	0.029	−0.378 **	0.006	−0.057 ***	0.000	−0.060 ***	0.000
Whether anyone in the family is a village level or above cadre (including village level cadres)	0.76	0.573			−0.025	0.792		
Whether a member of the farmers’ cooperative/association	−0.782	0.258			−0.013	0.706		
Market Expectations	1.486 **	0.004	1.368 **	0.002	0.091 ***	0.000	0.104 ***	0.000
Policy Advocacy	2.664 **	0.003	2.41 **	0.001	0.221 ***	0.000	0.220 ***	0.000
Policy Subsidies	1.873 **	0.035	1.575 *	0.051	0.040	0.295		
Calf racking cattle prices	0.066	0.246			0.001	0.791		
Geographical location	Control	Control	Control	Control	Control	Control	Control	Control
Constant	−7.732 *	0.052	−5.753 **	0.031	0.220	0.142	0.198	0.100

***, ** and * indicate passing the test at the significance level of 1%, 5% and 10%.

## Data Availability

The data presented in this study are available within the article.
